# Sorption competition with natural organic matter as mechanism controlling silicon mobility in soil

**DOI:** 10.1038/s41598-020-68042-x

**Published:** 2020-07-08

**Authors:** Thimo Klotzbücher, Christian Treptow, Klaus Kaiser, Anika Klotzbücher, Robert Mikutta

**Affiliations:** Soil Science and Soil Protection, Martin Luther University Halle-Wittenberg, Von-Seckendorff-Platz 3, 06120 Halle (Saale), Germany

**Keywords:** Biogeochemistry, Element cycles, Geochemistry

## Abstract

Growing evidence of silicon (Si) playing an important role in plant health and the global carbon cycle triggered research on its biogeochemistry. In terrestrial soil ecosystems, sorption of silicic acid (H_4_SiO_4_) to mineral surfaces is a main control on Si mobility. We examined the competitive sorption of Si, dissolved organic matter, and phosphorus in forest floor leachates (pH 4.1–4.7) to goethite, in order to assess its effects on Si mobility at weathering fronts in acidic topsoil, a decisive zone of nutrient turnover in soil. In batch sorption experiments, we varied the extent of competition between solutes by varying the amount of added goethite (α-FeOOH) and the Si pre-loading of the goethite surfaces. Results suggest weaker competitive strength of Si than of dissolved organic matter and ortho-phosphate. Under highly competitive conditions, hardly any dissolved Si (< 2%) but much of the dissolved organic carbon (48–80%) was sorbed. Pre-loading the goethite surfaces with monomeric Si hardly decreased the sorption of organic carbon and phosphate, whereas up to about 50% of the Si was released from surfaces into solutions, indicating competitive displacement from sorption sites. We conclude sorption competition with dissolved organic matter and other strongly sorbing solutes can promote Si leaching in soil. Such effects should thus be considered in conceptual models on soil Si transport, distribution, and phytoavailability.

## Introduction

Silicon (Si)—a major element within the earth's crust—has received substantial awareness by ecologists and biogeochemists during the last decade. It has been recognized as beneficial element for many plants, i.e., Si uptake can strengthen their resistance against a wide range of stresses, such as pests, diseases, and toxic metals. Moreover, the Si uptake by vegetation and the subsequent cycling of Si from plant litter is a major control on Si transport from terrestrial to aquatic ecosystems, where Si inputs determine algae growth, and thus, the fixation of atmospheric CO_2_^1^. Hence, on the regional and global scale, the Si cycle is tightly related to the cycling of carbon, which makes Si biogeochemistry a crucial topic within climate change research.

Silicon is taken up by plants from the soil solution, in which Si is mainly present in form of dissolved mono-silicic acid, and to a smaller extent in polymeric form^1^. The concentrations of Si in soil solution vary largely, depending on the composition and solubility of primary and secondary silicate minerals^1^, the plants’ Si intake, and the return of Si via plant residues into soil^2^. Hence, they are controlled by both, the natural site conditions as well as land management. Published values range between about 0.5 and 2000 µM, but mostly are between 100 and 500 µM^1^.

A number of laboratory experiments demonstrated that Si accumulates at the surfaces of Fe oxides, such as goethite, by forming Fe–O–Si bonds and polymerization products via Si–O–Si linkages^3–7^, a process that is discussed as a regulator of Si bioavailability by, for instance, preventing leaching of Si from topsoil^8,9^. Silicon bound at the Fe oxide surface can decrease the sorption of anions via competition for sorption sites, as demonstrated for anionic species of arsenic^10,11^, selenium^12^ and phosphorus (P)^13^. In turn, input of strongly sorptive components may cause a release of sorbed Si into solution^14–16^. Recent studies pointed at competitive sorption of Si and phosphate to Fe oxides as relevant for Si and P mobility in soil^17,18^, and the competition is known as a critical factor in water treatment efficiency^13^. There are also indications of sorption competition between Si and organic compounds^19^. Competition of Si with dissolved organic matter (DOM) and phosphate for sorption sites may have wide-ranging implications for ecosystem properties. Sorption of DOM to minerals is considered as major pathway in the formation of mineral-organic associations, and thus, an important factor for the long-term sequestration of organic carbon in soil^20^, while strong phosphate sorption might partly explain the widespread P limitation of plant growth^21^.

Here, we studied the sorptive interplay between Si, DOM, and P at goethite (α-FeOOH) surfaces, as goethite is the most relevant Fe oxyhydroxide in temperate soils. In particular, we quantified and compared the sorption of DOM, phosphate, and Si from two different natural forest floor solutions. Then, we tested how variable amounts of pre-sorbed Si affect the sorption of DOM and phosphate. The applied experimental conditions were representative for weathering fronts of acidic forest soils. We selected these conditions as (1) acidic soil conditions (i.e., pH of about 4–6) dominate under humid climate^22^, (2) the topsoil horizons typically are densely rooted, and thus important for nutrient turnover, (3) weathering induces formation of pedogenic Fe oxyhydroxides at high rates, (4) and the input of DOM and P via forest floor leachates is particularly high^23,24^, and thus, may be a central factor in Si mobilization and phytoavailability.

We hypothesized that pre-sorbed Si decreases the sorption of these components by occupying sorption sites, and thus, passivating the mineral surface’s reactivity towards anionic forest floor leachate components. Nevertheless, phosphate and part of the DOM are expected to be highly competitive sorbents, causing release of the pre-sorbed Si into solution. We specifically assessed Si–OH complexation by surface Fe, potentially hampering the surface complexation of organic ligands and phosphate, as the possible cause of sorption competition. The binding of Si was studied using sorption isotherm data and subsequent analysis of surface species by X-ray photoelectron spectroscopy (XPS).

## Results and discussion

### Silicon sorption to goethite surfaces

**Figure 1:**
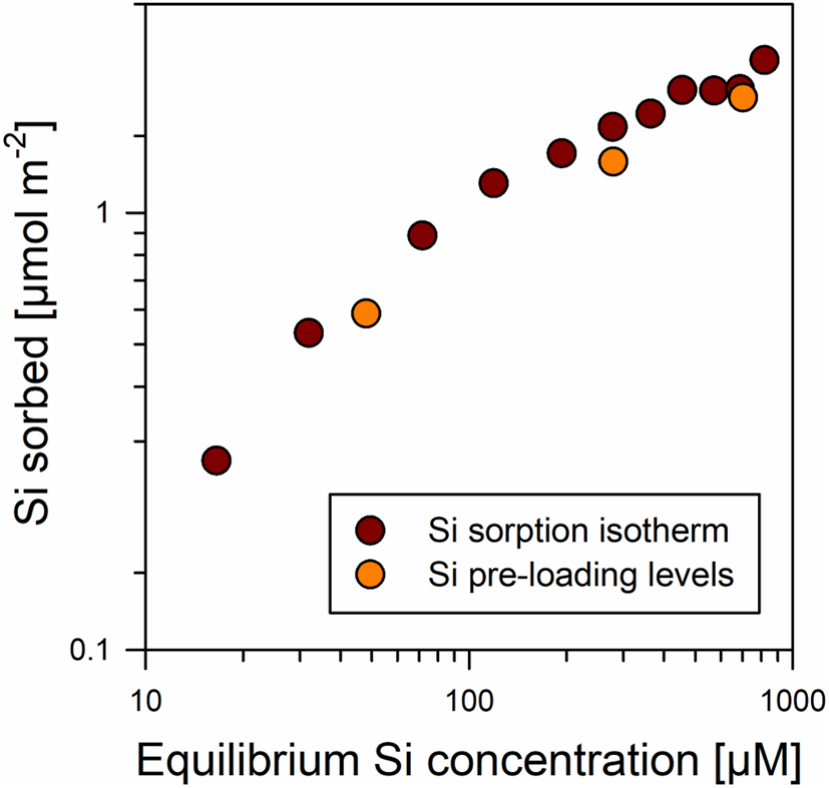
Relationship between equilibrium Si concentrations in solution and amounts of Si sorbed at the goethite surfaces for different experiments (Si sorption test and Si pre-loading of goethite for the experiments with forest floor solutions). Error bars (X- and Y-axis), denoting the standard deviation of replicates (n = 3), are smaller than the symbols. Data were plotted using SigmaPlot 11.0 from Systat Software, San Jose, CA.

In a first experiment, we examined Si sorption by comparing the relationship between dissolved Si concentrations in the equilibrium solutions after sorption and amounts of Si sorbed. Here, the dissolved Si concentrations varied between 0 and 819 µM, covering the common range of soil solution concentrations^1^. The relationship between Si in equilibrium solutions and amounts of Si sorbed (Si ‘loading’ levels) showed a logarithmic shape, i.e., the increases in Si loadings diminished at higher dissolved Si concentrations, yet, no clear plateau level indicating maximum surface coverage appeared (Fig. 1). The maximum Si surface loading (achieved at equilibrium concentrations of 819 µM Si) was 79 µmol Si g^−1^ or 2.2 µmol Si m^−2^. In contrast to our findings, other authors found steep increases in Si surface loadings at higher dissolved Si concentrations, which was explained by Si polymerization at Fe oxide surfaces (see data compilation and discussion in Song et al.^25^). In the study system at pH 4, the leveling-off of surface loadings at higher Si concentrations rather suggests limited availability of sorption sites for Si, which hints at Si sorption primarily via formation of Fe–O–Si bonds. It should be noted that presence of H_2_CO_3_ in solutions may have reduced Si sorption at pH 4^26^. In case of such an interference, it should be most pronounced at small Si concentrations since the H_2_CO_3_ concentration does not depend on Si level. Based on equilibrium calculations (Visual MINTEQ; ver. 3.1; KTH, SEED, Stockholm, Sweden), we estimated that the H_2_CO_3_ concentration in solutions were ~ 12 µM, which is in the range of the lower Si concentrations used (i.e., 4–72 µM Si for the three initial solutions with the lowest Si concentrations). Yet, the finding that the sorption envelope showed no deviation from the typical L shape at smaller Si concentrations (Fig. 1), suggests no or negligible repression of Si sorption at smaller concentrations.

### Pre-loading of the goethite with silicon

On basis of the data presented above, we selected the levels of Si pre-loading for the subsequent experiments with natural forest floor solutions. The results on Si sorption during the pre-loading procedure matched the results of the prior sorption experiment (Fig. 1), indicating good experimental reproducibility. Rinsing the goethite for removal of residual salts removed weakly bound Si from the surfaces; the losses averaged 5% of the initial surface-bound Si. The final Si loading levels of the samples (after sorption and washing) were 20, 44, and 60 µmol Si g^−1^ or 0.5, 1.1, and 1.7 µmol Si m^−2^, which represented 25%, 56%, and 76% of the maximum Si surface loading achieved in the prior sorption experiment. No higher loading levels were used to avoid Si polymerization at the surfaces, and thus, other Si binding types being involved in the sorption competition. The Si pre-loading levels of samples are denoted in the following with ‘low’, ‘medium’, and ‘high’.

The pre-loading of the goethite with Si had no clear effect on the SSA, which averaged 38.4 m^2^ g^−1^ for all used goethite samples, including the one without Si loading (Table 1). This value was within the range of values reported for goethites^27,28^. Similarly, we found no large differences in the ζ potential (pH 4) between the Si pre-loading levels, which equaled on average 40.8 mV (Table 1). Hence, surface area and charge of the different Si pre-loading levels were no factors in the sorption experiments with natural forest floor solutions.

**Table 1:** Specific surface area (SSA) and ζ potential of the goethite samples with different Si pre-loading levels. Given are average values and, in brackets, standard deviations of n = 3 (SSA) or n = 5 (ζ potential) replicated analyses.

	No Si pre-loading	Low Si pre-loading	Medium Si pre-loading	High Si pre-loading
SSA (m^2^ g^−1^)	35.4 (2.5)	41.5 (8.1)	40.3 (3.0)	36.2 (2.0)
ζ potential at pH 4 (mV)	35.0 (1.0)	41.9 (0.7)	42.5 (1.1)	44.2 (1.6)

**Figure 2:**
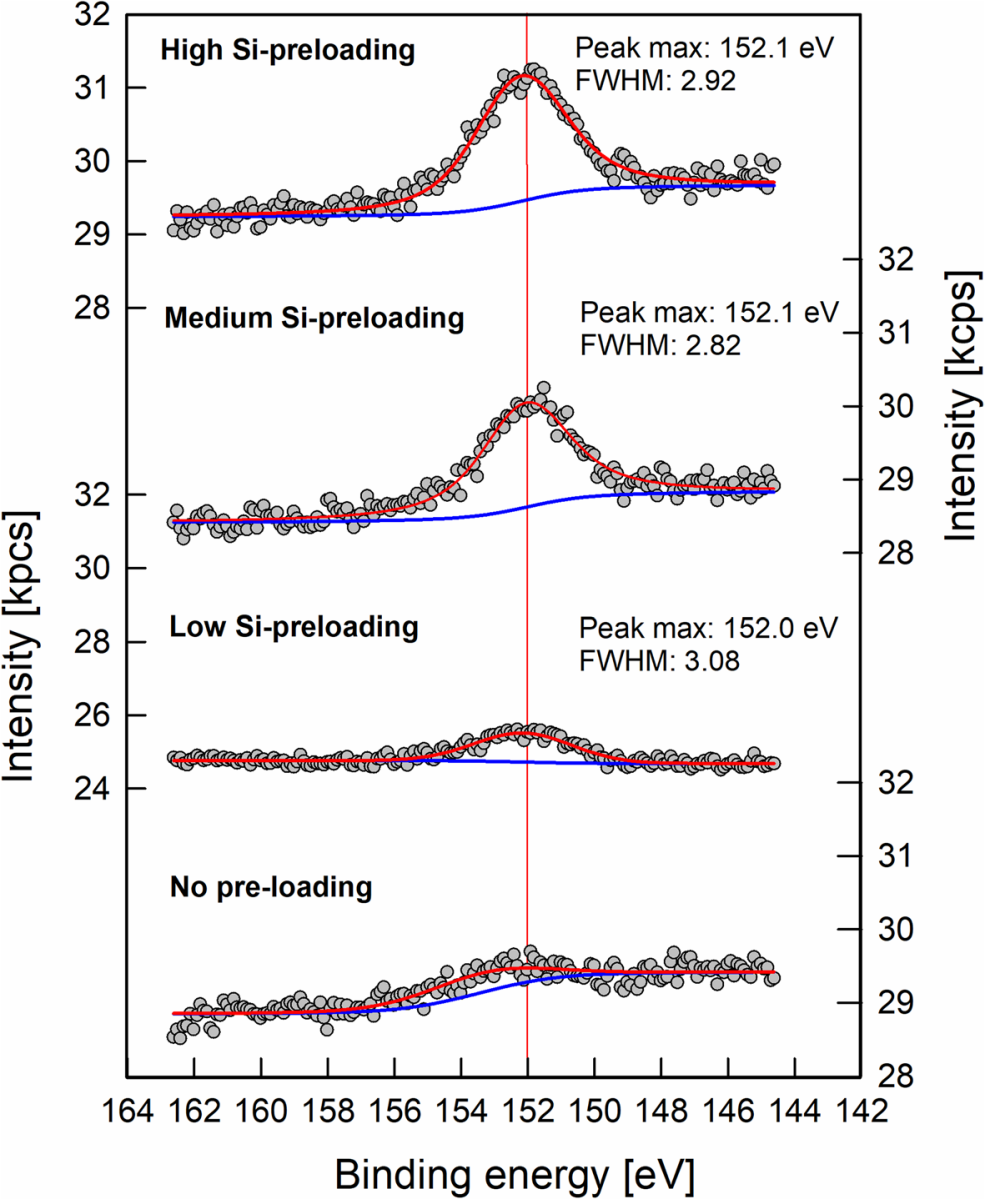
Examples of XPS spectra of the Si 2*s* region for goethite with variable Si pre-loadings prior to the sorption experiments with forest floor solutions. The vertical red line at 152.0 eV highlights that there were no differences in peak position between pre-loading levels, thus indicating the prevalence of monomeric Si. Data were plotted using SigmaPlot 11.0 from Systat Software, San Jose, CA.

The Si/Fe elemental ratios calculated on basis of XPS data were 3.9∙10^−2^ ± 1.4∙10^–2^, 6.0∙10^–2^ ± 1.2∙10^–2^, and 6.6∙10^–2^ ± 4.1∙10^–3^ for the ‘low’, ‘medium’, and ‘high’ Si pre-loading levels. According to Swedlund et al. (2011)^29^, shifts of the XPS Si 2*s* signal by up to 1 eV towards higher binding energies are indicative of increasing contribution of Si–O–Si bonds, and thus, increasing surface polymerization. We did not find such shifts in binding energy with increasing Si pre-loading (Fig. 2). Instead, the binding energies were in the range reported for predominately Fe–O–Si bonds^29^. In particular, signal maxima were at 152.3 ± 0.4 eV, 152.2 ± 0.3 eV, and 152.0 ± 0.2 eV for the ‘low’, ‘medium’, and ‘high’ Si pre-loading levels (average values and standard deviation of three analysis positions). The XPS results thus support the data from the Si sorption experiment, suggesting that Si at the goethite surface was primarily coordinated in Fe–O–Si bonds, and that no Si polymers were present. The lack of detectable Si polymerization at the surfaces (i.e., XPS data and Si sorption curve) may be explained by the short reaction time of 24 h. While sorption of monomeric Si to surficial Fe atoms is relatively fast and typically reaches its maxima after minutes to a few hours^6^, surface polymerization is slower; it starts later and can continue over long periods of time^6,11^. In studies on the sorption of Si to hematite, even after 210 days, no equilibrium was reached due to progressing surface polymerization^11^. Also in 60-days laboratory experiments with soil material, Si sorption increased until the end of the observation period^30^. Hence, our data apply to sorption phenomena in relatively fast draining soil material. Due to continuous percolation, contact times between soil solution and the solid phases are often short in many topsoils under humid climate. For example, in an acidic forest soil (Dystric Cambisol) in Northern Germany, water fluxes at 10 cm soil depth mirrored well the precipitation events, and even small events resulted in percolating water; it was estimated that water fluxes at 10 cm soil depth represented 70% of the throughfall^31^. An additional factor of Si polymerization (which was not assessed herein) may be the morphology of goethite samples, which determines relative distribution of surface types; more particularly, the work of Song et al.^25^ suggests that (021) crystal faces of goethite promote polymerization, whereas (110) faces do not allow polymerization.

### Initial properties of the natural forest floor solutions

The pH value was slightly higher in the Bad Brückenau than in the Mitterfels solution (4.7 vs. 4.1; Table 2), and this difference might have contributed to the differences in sorption processes discussed below. It has been demonstrated for the pH range studied here that the amounts of Si sorbing to Fe oxide surfaces increase with increasing pH^3^, while the sorption of DOM typically decreases^32^.

**Table 2:** Initial properties of the two forest floor solutions.

Solution source	pH	DOC (mM)	SUVA_280_ (l mmol^−1^ C cm^−1^)	Total P (µM)	Phosphate-P (µM)	Si (µM)
Bad Brückenau	4.7	3.9	0.36	1.5	0.04	237.4
Mitterfels	4.1	1.5	0.41	12.7	0.33	41.6

The Bad Brückenau solution had higher initial DOC and dissolved Si concentrations (Table 2), and the initial molar C/Si ratios in the solutions were 16.5 (Bad Brückenau) and 36.6 (Mitterfels). Initial SUVA_280_ values were similar for the two solutions (Table 1), suggesting similar relative contributions of aromatic moieties to DOM. We determined the aromaticity of DOM because aromatic components of forest floor solutions have a high affinity to bind to Fe oxide surfaces^33^. Given the higher molar C/Si ratio and similar SUVA_280_ values, we expected that the DOM of the Mitterfels solution is more competitive against Si for sorption sites than those of the Bad Brückenau solution.

The total P and phosphate-P concentrations were higher in the Mitterfels than in the Bad Brückenau solution (Table 2). In both solutions, the initial phosphate-P concentrations made only about 3% of the total P concentrations, suggesting most P to be present in organic form. Moreover, as phosphate-P concentrations made less than 1% of the dissolved Si concentrations, it played only a minor role as a competitor for sorption sites in the present study.

### Sorption of forest floor solution constituents to goethite without Si pre-loading

**Figure 3:**
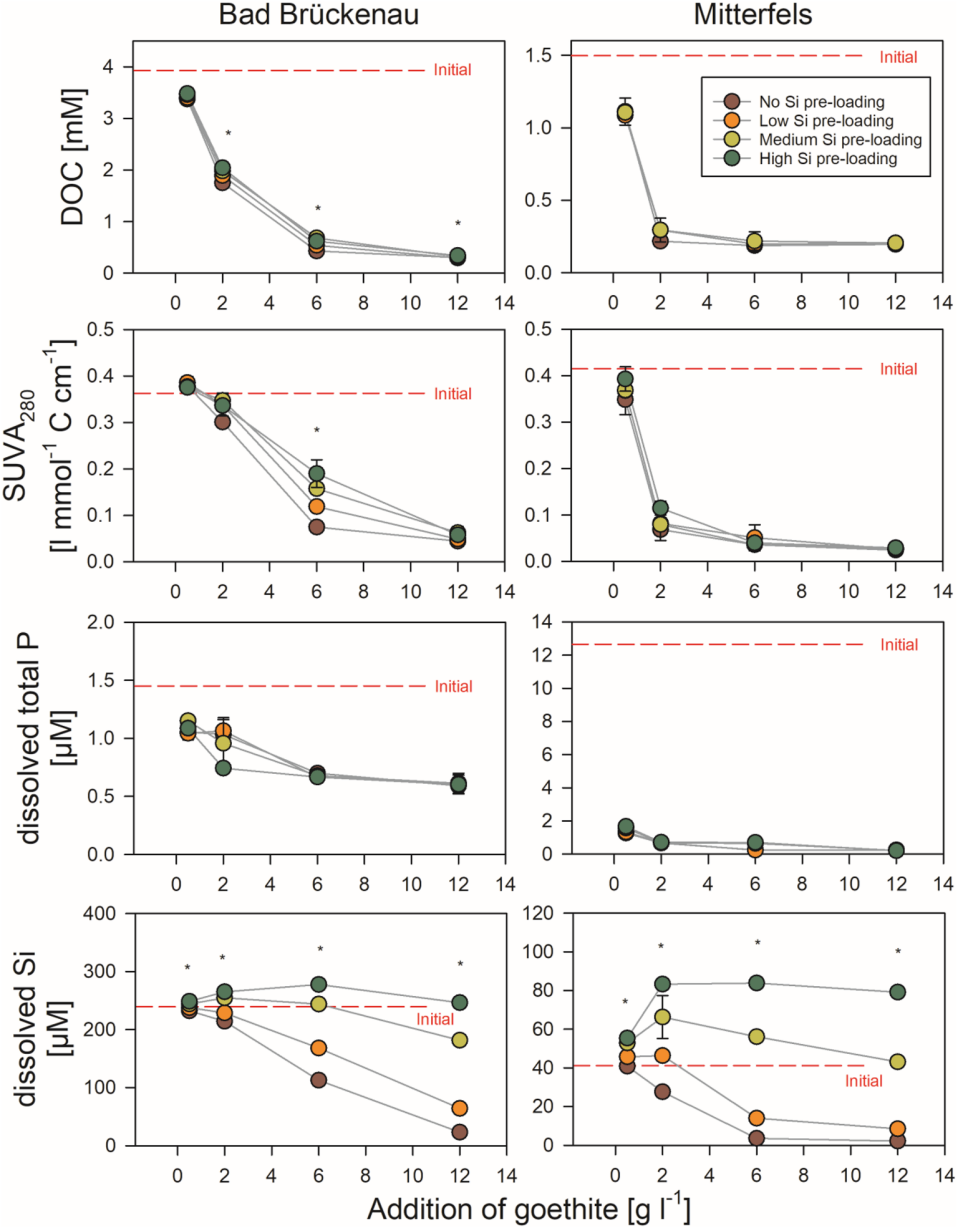
Changes in composition of the two forest floor solutions upon different additions of goethite with and without Si pre-loadings. The error bars indicate the standard deviation of experimental replicates; stars indicate significant differences between treatments (*p* < 0.05; ANOVA on ranks), i.e. at least two treatments differ significantly from each other. SUVA_280_ indicates the specific UV absorbance measured at 280 nm and normalized to DOC. Data were plotted using SigmaPlot 11.0 from Systat Software, San Jose, CA.

**Figure 4:**
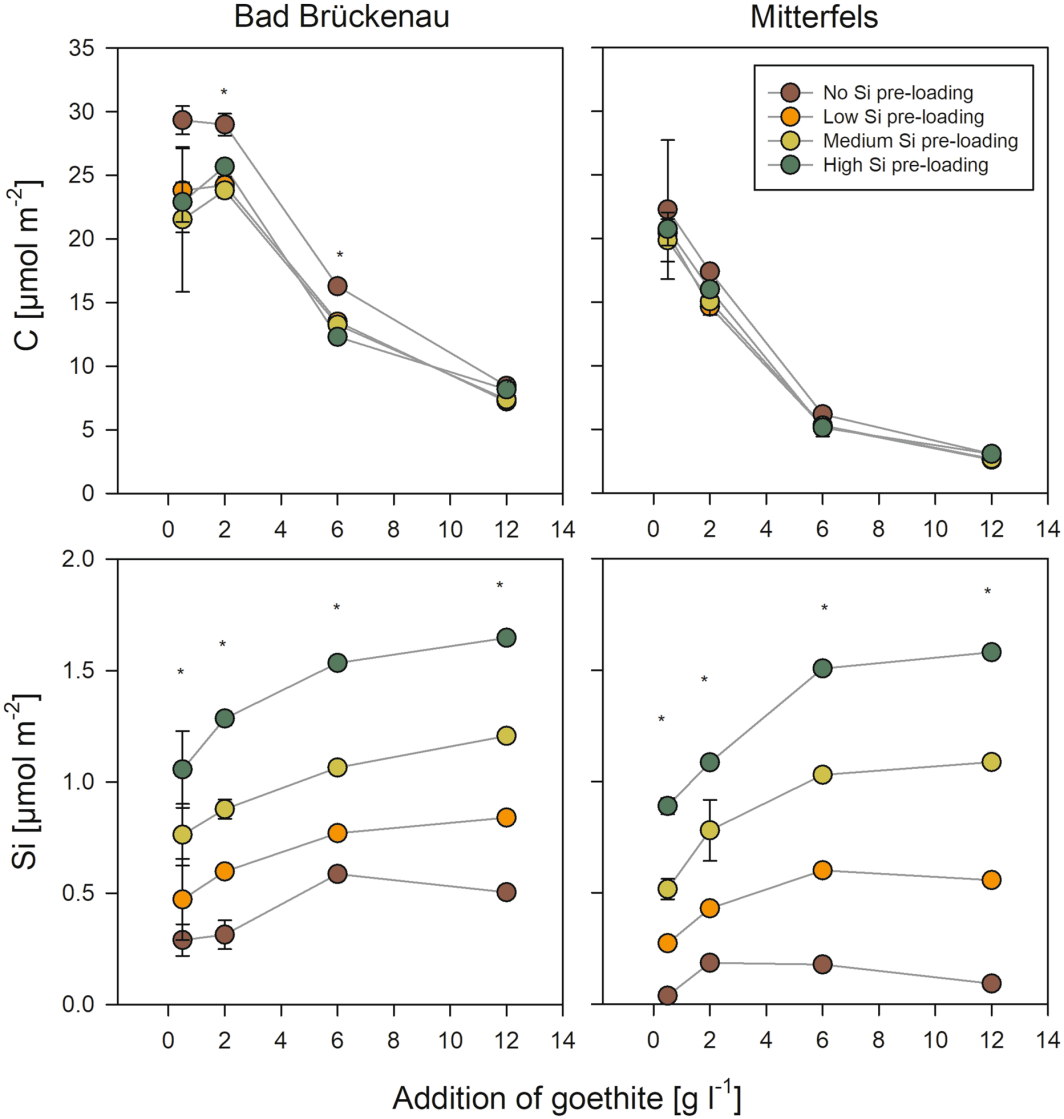
Amount of organic C and Si at goethite surfaces after interaction with the two forest floor solutions upon different additions of goethite with and without Si pre-loadings. The error bars indicate the standard deviation for experimental replicates; stars indicate significant differences between treatments (*p* < 0.05; ANOVA on ranks), i.e. at least two treatments differ significantly from each other. Data were plotted using SigmaPlot 11.0 from Systat Software, San Jose, CA.

The DOC concentrations decreased with increasing addition of goethite for both forest floor solutions (Fig. 3). The maximum decrease was by 93% for the Bad Brückenau solution and by 88% for the Mitterfels solution, re-confirming the well-established strong DOC sorption by goethite^34,35^. At goethite additions ≥ 6 g l^−1^ for the Bad Brückenau solution and ≥ 2 g l^−1^ for the Mitterfels solution, no additional DOC was adsorbed. This suggests that the portions of organic compounds in the solutions capable to bind were limited and already completely sorbed once a certain level of sorption sites was offered. In turn, the C loading of the goethite surfaces decreased with increasing goethite addition, i.e., when increasing amounts of sorption sites offered (Fig. 4). Maximum values were 1.0 mmol g^−1^ (0.03 mmol m^−2^) for the Bad Brückenau solution and 0.8 mmol g^−1^ (0.02 mmol m^−2^) for the Mitterfels solution. For the Bad Brückenau solution, the C loadings were similarly high for goethite additions of 0.5 and 2 g l^−1^, suggesting that the surfaces were ‘saturated’ with organic matter, i.e., all sorption sites available for potentially sorbing DOM components were occupied, leaving compounds with weaker sorption affinities in solution. The C saturation level observed for the Bad Brückenau solution was at the lower end of values reported in studies using a comparable experimental design^34–36^.

Decreases in SUVA_280_ values upon goethite additions indicated selective removal of aromatic components from the forest floor leachates (Fig. 3), which is consistent with literature reports^33^. The aromaticity of DOM, typically, is higher in case the DOM derives from more decomposed plant litter (as stored, e.g., in lower parts of forest floor layers) than from fresh litter, probably due to the higher contribution of soluble aromatic products of lignin depolymerization released during later stages of litter decomposition^37^. These oxidized components are rich in carboxyl groups that can form strong inner-sphere complexes at the surfaces of metal oxide phases, particularly at lower pH values (as in our study) when many surface hydroxyl groups at the oxide surfaces are protonated and positively charged^36^.

Addition of goethite without Si-preloading decreased the concentrations of total P and phosphate in both solutions. Even the smallest addition of goethite caused a decrease in phosphate concentrations to values below detection limit (not shown). This corresponded to the well-known high sorption affinity of phosphate to Fe oxide surfaces under acidic conditions^38^. The decreases in total P concentrations were up to 59% (Bad Brückenau solution) and 98% (Mitterfels solution) of the initial values (Fig. 3). Especially for the Bad Brückenau solution, the relatively high amount of P remaining in solution demonstrates that some of the P-containing organic compounds can have a low sorption affinity for goethite surfaces even under acidic conditions. This finding agrees well with results from previous sorption experiments and field studies^39, 40^.

Addition of low amounts of goethite without Si pre-loading (0.5 g l^−1^) resulted in no or only small decreases in dissolved Si concentrations (< 2%; Fig. 3), which indicates a lower sorption affinity of Si than of other components of the solutions. At higher goethite additions, with more sorption sites being available, considerable decreases in dissolved Si concentrations were found; it decreased by 90% in the Bad Brückenau and by 95% in the Mitterfels solution (Fig. 3). The maximal Si loadings achieved were 21 µmol g^−1^ or 0.6 µmol m^−2^ (Bad Brückenau solution) and 7 µmol g^−1^ or 0.2 µmol m^−2^ (Mitterfels solution), respectively. The Si loadings showed the opposite trend as the C loadings; they increased with increasing goethite addition (Fig. 4). This also points at a lower sorption affinity of Si than of C, i.e., Si only sorbs to a larger extent when excess sorption sites are available.

Moreover, for goethite additions of up to 2 g l^−1^ (Mitterfels solution) or 6 g l^−1^ (Bad Brückenau solution), the molar ratios between DOC and dissolved Si decreased with increasing goethite input (Mitterfels solution: from 27 to 8; Bad Brückenau solution: from 15 to 4). At higher goethite additions, they increased again (Mitterfels solution: from 8 to 92; Bad Bückenau solution: from 4 to 13). This suggests that Si was only sorbed when all strongly sorbing organic matter components were already removed from solution and still some sorption sites were available.

### Sorption of forest floor solution constituents to goethite with Si pre-loadings

Effects of pre-loaded Si on changes in DOC (Fig. 3) as well as on C loadings of the goethite surfaces (Fig. 4) were rather small compared to the effects of adding different amounts of goethite. For the Mitterfels solution, no significant differences between Si pre-loading levels were found, and for the Bad Brückenau solution the difference were significant only in few cases. For these cases, our data indicate that the Si pre-loadings reduced the sorption of DOM components. This reduction (as calculated by changes in DOC concentrations) made up to 13% of the amounts of DOC sorbed to goethite without Si pre-loading.

**Figure 5:**
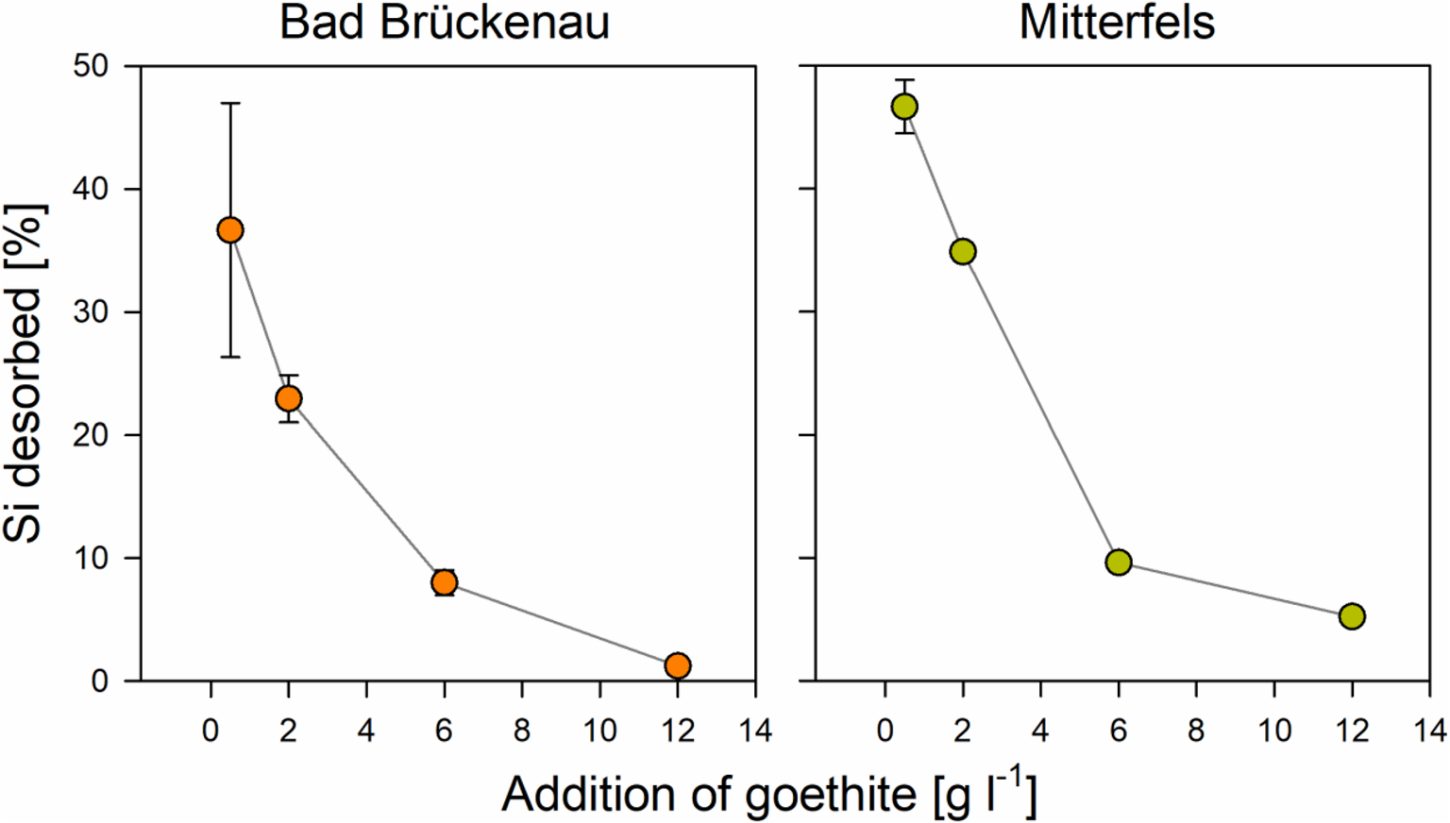
Percentage of Si desorbed from the goethite surfaces with high Si pre-loading due to interactions with the two forest floor solutions as a function of the goethite amounts added (data for other Si-preloading levels are not shown because less pronounced or no desorption occurred); the error bars indicate the standard deviation for experimental replicates. Data were plotted using SigmaPlot 11.0 from Systat Software, San Jose, CA.

One possible reason for the small effects of the Si pre-loading on the C loadings is the seemingly weak sorption affinity of Si for sorption sites. We found that Si was released into the solutions from goethite with medium and high Si pre-loadings, causing increases in dissolved Si concentrations by up to 31 µM (Bad Brückenau) and 42 µM (Mitterfels; Fig. 5). The portion of initially surface-bound Si released into solution showed a decrease with increasing availability of sorption sites (i.e., with increasing goethite addition; Fig. 5). This finding clearly indicates that Si was displaced from the goethite surface by compounds with a higher sorption affinity, such as phosphate and DOM components. The maximum portions of Si released were 37% (Bad Brückenau solution) and 53% (Mitterfels solution) of the initial Si present at the surfaces. Hence, a large fraction of the pre-sorbed Si was not removed by reaction with the forest floor solutions, and as a consequence, the Si loading at the goethite surfaces after the sorption experiments differed significantly between samples with different initial Si loadings (Fig. 4). These calculations suggest that not all results can be explained by sorption competition, and that additional factors need to be considered for explaining the small effect of Si pre-loadings on the sorption of other forest floor leachate components. One could be that maximum Si coverage used in the experiments was only 76% of the maximum Si surface loading achieved in the prior sorption experiment. An incomplete Si surface coverage of the goethite was also indicated by the small surface Si/Fe ratios determined by XPS. Data obtained in our laboratory within another study showed that the studied goethite binds up to about 90 µmol ortho-phosphate g^−1^ until the plateau level is reached; this is about 1.5 times the amounts of Si bound at the goethite surfaces at the highest Si pre-loading levels used here. Hence, a considerable part of the other forest floor leachate components might have been bound to sorption sites not occupied by Si, and thus, competition and subsequent displacement of Si become only relevant for the small goethite additions, where available sorption sites were more limited. In addition, the only partial competitive sorption theoretically may hint at differing preferences of Si and organic compounds for certain parts of the goethite surface.

### Implications

**Figure 6:**
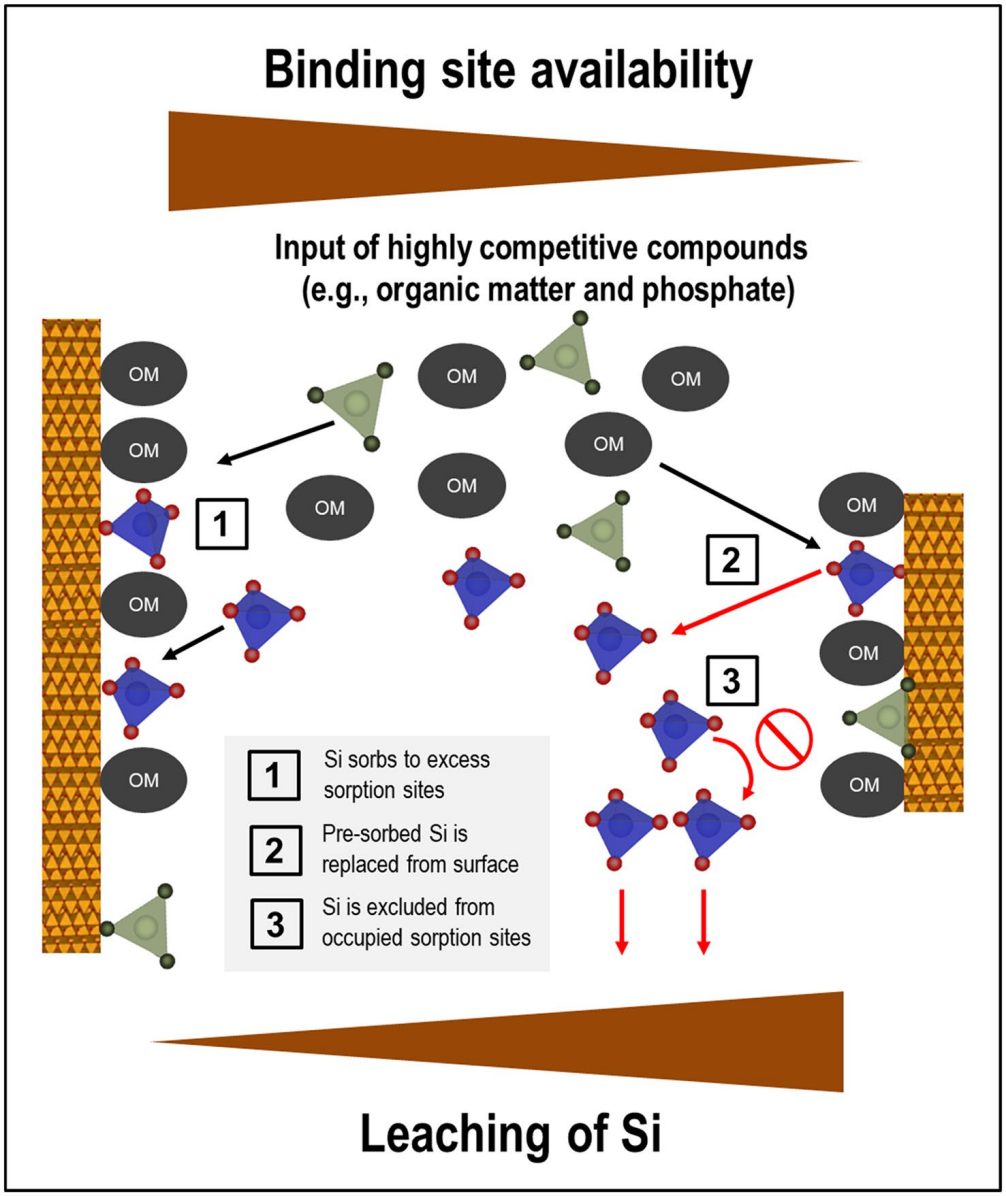
Nature and implications of sorption competition between Si (blue symbols), organic matter (dark grey symbols), and phosphorus (green symbols) at the variable-charge surfaces of pedogenic minerals.

We present for the first time data on competitive interactions between Si and other dissolved components of natural forest floor solutions, such as DOM and phosphate, at goethite surfaces. Overall, our experiments with goethite with and without Si pre-loading suggest that under acidic conditions the competitive strength of Si for sorption sites is low when compared to that of DOM and phosphate (Fig. 6). This implies that in acidic topsoil environments, where input of competitive organic and inorganic components with forest floor leachates is high, retention and accumulation of Si (either from mineral weathering or entering with forest floor leachates) at the surfaces of newly formed hydrous Fe oxides or other similar mineral phases should be comparatively little, while mobility of Si should be high. Nevertheless, we observed significant Si sorption at higher goethite additions, i.e., when more sorption sites were available, a situation that is more typical for subsoils. Hence, sorption competition should be a major factor affecting the partitioning of Si between the dissolved and solid phase within soil profiles. Future work needs to address how this determines major aspects of the biogeochemical Si cycle, including Si phytoavailability, formation of secondary silicate minerals as well as export of Si from soil into the hydrosphere.

## Methods

### Goethite synthesis

Goethite was produced as outlined by Kaiser and Guggenberger^35^. Sample purity was verified by X-ray powder diffraction analysis using Cu Kα radiation from 5° to 70° 2θ with a step size of 0.02° 2θ, and time steps of 20 s (X’Pert^3^ Powder, PANalytical, Alemelo, The Netherlands). The specific surface area (SSA) was determined by N_2_ gas adsorption at 77 K, based on 11 adsorption points in the relative pressure range of 0.05–0.3, and applying the Brunnauer–Emmett–Teller (BET) equation (Autosorb iQ MP, Quantachrome GmbH, Odelzhausen, Germany). Moisture was removed before measurement by degassing the sampling at 60 °C for 6 h.

### Silicon sorption and pre-loading the goethite with Si

All Si solutions were prepared from stock solution of mono-silicic acid (1,000 mg Si l^−1^ in 2% NaOH; Certipur, Merck, Darmstadt, Germany). In order to prevent solution phase polymerization, the stock solution was diluted by at least 25 times before adjusting the pH of all Si solution to 4 (close to the pH of the forest floor leachates) with HCl, and the ionic strength to 0.015 M with NaCl.

Silicon sorption experiments were conducted, using suspensions with 1.82 g l^−1^ goethite and twelve different initial Si concentrations (4–1,060 µM Si; pH 4; n = 3 experimental replicates). The suspensions were agitated in polypropylene bottles on a horizontal shaker at 200 rpm for 24 h at 22–24 °C, and then centrifuged at 8,525 g for 35 min. The supernatant was passed through 0.1-µm polyethersulfone membranes (Supor-100, Pall Science Corp., Ann Arbor, MI, USA) before analysis for Si by inductively coupled plasma–optical emission spectroscopy (ICP-OES; Ultima 2, Horiba Jobin–Yvon, Longjumeau, France). The amounts of Si sorbed to the goethite equaled the difference of the initial Si concentration and the equilibrium Si concentrations after 24 h.

Based on the results of these sorption experiments, we selected Si pre-loading levels of the goethite for the experiments with forest floor solutions. For Si pre-loading, suspensions with 1.82 g l^−1^ goethite and Si concentrations of 0, 100, 400, and 900 µM Si were prepared (pH 4), and treated as described above for the Si sorption experiment. In order to remove soluble salts from the goethite samples, they were re-suspended several times in doubly deionized water, followed by centrifugation at 8,525 g for 35 min and discarding of the supernatant until the electric conductivity of the supernatant was ≤ 50 µS cm^−1^. The Si-loaded goethite samples were then freeze-dried.

### Analysis of Si-loaded goethite surfaces

The Si binding environment on the Si-loaded goethite as well as Si-to-Fe element ratios were assessed using XPS (Axis Supra instrument, Kratos Analytical, Manchester, UK). The analyses were obtained for freeze-dried goethite powders that were mounted on double-sided adhesive carbon tape. For each sample, we obtained detail scans of (1) the C 1*s* signal (between 274 and 300 eV) for correcting the binding energies of all analyses, (2) the Si 2*s* signal (between 142 and 160 eV) to deduce the Si binding environment, and (3) the region between 4 and 160 eV in order to calculate Si-to-Fe element ratios from the Si 2*s* and Fe 3*p* peaks. Three measurement positions (each 300 × 700 µm) per powder sample were analyzed. Settings were: emission current of 25 mA (detail scans), step size of 0.1 eV (detail scans), and up to five sweeps (the number was less in case a quality control value was reached that was calculated by the software on basis of differences in peak height and peak to peak variation between sweeps). Data were evaluated using the ESCApe software (vs. 1.2.0.1325; Kratos Analytical, Manchester, UK). Peak maxima were obtained from first derivatives of the signal curves (using five point stencil differentiation) to test for possible Si polymerization at the surfaces.

Electrophoretic mobility measurements were carried out at room temperature using the laser Doppler electrophoresis approach (Nano-ZS, Malvern Panalytical, The Netherlands). Goethite samples were suspended in ultrapure water adjusted to pH 4, and five measurements per sample were conducted within less than seven minutes. The ζ potential was derived from the electrophoretic mobility data using the Smoluchowski equation. The SSA of the pre-loaded goethite was determined as described above.

### Forest floor solutions

We used two solutions collected by lysimeters installed beneath the forest floor in two ~ 140 year-old beech forests. One is on Dystric Skeletic Cambisols with mull-type forest floor (“Bad Brückenau”), the other on Hyperdystric Chromic Folic Cambisols with a moder-type forest floor (“Mitterfels”). More details on the study sites are given in Lang et al.^23^. The solutions were collected in October 2017, transported cooled into the laboratory, and filtered through 0.1-µm polyethersulfone filters (Supor-100) before use. The initial solutions were analyzed for dissolved organic C (DOC), using combustion catalytic oxidation (multi N/C 3,100, Analytik Jena, Jena, Germany), total dissolved Si and P (by ICP-OES, Ultima 2), phosphate (using a continuous flow analyzer, SAN++, Skalar, Breda, Netherlands), and the UV absorbance at 280 nm (SPECORD 250 Plus, Analytik Jena, Jena, Germany). The UV absorbance was normalized to the DOC concentrations to obtain the specific UV absorbance (SUVA_280_), which is an estimate of the contribution of aromatic compounds to DOM^41^.

### Sorption experiments with forest floor leachates and goethite with and without Si pre-loadings

The sorption experiments were conducted in triplicates by adding variable amounts of goethite (0.5, 2.0, 6.0, and 12.0 g l^−1^) without or with varying Si pre-loading levels to each of the forest floor leachates. The additions were chosen on basis of information about the capacity of goethite (without Si pre-loading) to bind DOM obtained in previous experiments^35^. Consequently at the lowest additions, the sorption capacity was assumed to be almost completely exhausted, causing maximum competition among for sorption sites; the extent of competition should gradually decrease with increasing goethite additions.

Sorption experiments were conducted for 24 h, during which the suspensions were agitated in polyethylene bottles on a horizontal shaker at 200 rpm at 22–24 °C. Then, the goethite was settled by centrifugation (8,525 g for 35 min) and the supernatant solutions were filtered through 0.1-µm polyethersulfone filters (Supor-100) before analyses for DOC, phosphate-P, total P, SUVA_280_, and total Si as described above for the initial solutions. Amounts of C, P, and Si sorbed to the mineral surfaces were calculated via changes in the respective concentrations in solutions during the experiments.

### Statistical methods

The ANOVA on ranks method of the SigmaPlot 11.0 software (Systat Software, San Jose, CA) was used to test for statistically significant effects of the Si pre-loading levels on concentrations of DOC and phosphate and on the SUVA_280_ in solutions as well as on C and Si loadings at the goethite surfaces after the sorption experiment. The non-parametric method was chosen as the underlying assumptions of one-way ANOVA (normal distribution of data groups, as tested by the Shapiro–Wilk test, and equality of variances, as tested using Levene’s test) were in many cases not met.

### Data availability statement

The datasets generated during and/or analyzed during the current study are available from the corresponding author on reasonable request.
